# The Reliability of Using a Laser Device to Assess Deceleration Ability

**DOI:** 10.3390/sports7080191

**Published:** 2019-08-09

**Authors:** Jonty Ashton, Paul A. Jones

**Affiliations:** 1 Department of Sport, Health and Exercise Sciences, University of Hull, Hull HU6 7RX, UK; 2 School of Health & Society, Directorate of Psychology and Sport, University of Salford, Salford M6 6PU, UK

**Keywords:** performance, sprint, change of direction, rugby, agility

## Abstract

An important component of change of direction speed is the ability to decelerate. Objective methods to examine this quality have been rarely reported in the literature. The aim of this study was to investigate the within- and between-session reliability (intraclass correlation coefficients (ICC), coefficient of variation (CV), standard error of measurement (SEM) and smallest detectable difference (SDD)) of using a laser Doppler device (LAVEG—LAser VElocity Guard) to quantify deceleration ability in 20 amateur rugby union players. Each player performed one familiarisation and two experimental sessions (seven days apart) consisting of three maximal 15 m sprints from a standing start, with an immediate deceleration to a complete stop upon hearing an audible cue at the 15 m mark. Deceleration was evaluated by determining the distance required to decelerate to 75%, 50%, 25% and 0% (‘stopping distance’) of the velocity achieved at 15 m of the maximal sprint. Within-session relative reliability was moderate to good (ICC = 0.64–0.83) with borderline acceptable variation (CVs = 10.51%–16.71%) across all variables. Between-session reliability reported good to excellent relative reliability (ICC = 0.79–0.93) with acceptable absolute reliability, particularly for stopping distance (SEM: 6.54%; SDD: 9.11%). The assessment shows promise as a method to quantify deceleration ability in athletes.

## 1. Introduction

Rapid deceleration is observed in a variety of field- (i.e., soccer, rugby, American football, etc.) or court- (i.e., netball, basketball and racket sports) based sports when requiring to stop or as a precursor to a change of direction manoeuvre [1]. In court-based sports, due to playing area dimensions athletes may be required to decelerate rapidly over short distances, whereas in field sports such as soccer and rugby, players may be required to decelerate from varying velocities and over a variety of distances [1]. Time–motion analysis in soccer (professional reserve, U21 and U18 level) has revealed 43–62 high-intensity decelerations (≤−3 m·s^−2^) per game are performed [2,3]. Whereas in the first and second halves of professional rugby union matches, 28.3 ± 10.6 and 25.9 ± 12.1 decelerations (≤−3 m·s^−2^) per half have been reported, respectively [4]. Furthermore, playing position in both soccer [3] and rugby union [5] has been shown to influence the number of decelerations (≤−3 m·s^−2^) performed, suggesting that distinct playing boundaries in field-based sports influence the intensity and duration of decelerations during match play [1].

Despite the importance of deceleration in sport, until recently there has been limited attention in the literature to evaluate this quality. Biomechanical studies have examined deceleration as a precursor to a change of direction manoeuvre, identifying the presence of distinct kinematic and kinetic differences between the ‘final’ plant step and steps preceding the change of direction manoeuvre [6,7]. Further studies have indicated the importance of kinetic parameters during the penultimate foot contact (second-to-last foot contact prior to the directional change) for both performance [8,9] and associated injury risk factors [7]. Dos’Santos et al. [8] reported that higher braking forces in the penultimate step of a change of direction manoeuvre related to faster change of direction performance, whereas Jones et al. [9] found that the penultimate step was an important step to regulate approach velocity prior to a 180° turn for faster individuals. Jones et al. [7] also found greater average horizontal braking forces during penultimate foot contact during cutting and greater average horizontal braking forces during penultimate compared to final contact of pivoting was associated with lower peak knee abduction moments (a knee joint load associated with increasing anterior cruciate ligament strain during change of direction manoeuvres [7]) during the final ‘plant’ step. Collectively, these studies highlight the importance of deceleration during steps prior to change of direction manoeuvres.

Change of direction speed or ability is defined as the ‘the ability to decelerate, reverse or change movement direction and accelerate again and is considered pre-planned’ [10]. This quality evaluates the physical and technical abilities to effectively change direction and is an underpinning quality of agility (defined as, “a rapid and accurate whole-body movement with a change of velocity, direction or movement pattern in response to a stimulus” [10]). Despite a lot of attention in methods to evaluate both change of direction speed and agility, little attention has been paid to develop methods in order to evaluate deceleration, even though, as mentioned previously, an important component of change of direction speed is the ability to decelerate. 

The first reported method for assessing an athlete’s ability to decelerate from a sprint was suggested by Plisk [11]. Plisk’s method [11] involved counting the number of steps to a complete ‘stop’ from sprints of varying intensity (100%, 75% and 50%). Plisk [11] suggested that this method can be utilised to both develop and evaluate an athletes’ deceleration ability. However, no literature is available to support this method, and may be considered largely subjective (such as quantifying approach velocity). Naylor and Greig [12] evaluated stopping distance from a 10 m maximal sprint using a standard tape measure to determine distance from the finishing line to the posterior heel of the stationary participant on the complete stop. The test was used in a study to explore the interaction between a battery of physical and cognitive profiling tests against four common agility tests, thus, did not report any validity or reliability of the method. Although, an improvement upon earlier methods [11], the method lacks an objective quantification of entry velocity into the deceleration zone, and the ability to measure further characteristics of deceleration. Harper et al. [13] utilised video analysis using a low sampling rate of 50 Hz, to determine and calculate maximal linear deceleration distance and time to stop from a 20 m maximal sprint. They determined approach velocity by calculating the velocity within the final 1 m of the approach to the deceleration zone. This approach has the advantage of providing the ability to also measure certain characteristics of deceleration such as stride length and frequency, number of steps, trunk and lower limb joint angles upon deceleration efforts. 

More recently researchers have used laser Doppler technology (LAVEG— LAser VElocity Guard) to quantify stopping distances [14]. This study measured instantaneous velocity during acceleration and (immediate) deceleration efforts within set distances of 5, 10, 15 and 20 m determining maximum velocity, stopping distances and percentage of maximum velocity based on a prior 30 m sprint test. A ‘deceleration gradient’ was also calculated for each subject using the mean peak velocity and stopping distance data for the 5 and 10 m trials. Equations were generated linking % maximum velocity attained with acceleration distance (R^2^ = 0.961) and stopping distance (R^2^ = 0.851), which could then be used to set realistic conditions for acceleration–deceleration drills. The use of laser Doppler devices provides an avenue to objectively quantify deceleration capabilities. However, no research has yet evaluated the reliability of previously used deceleration tests [12,13,14] and no research has considered using the laser Doppler device in this regard. Therefore, the aim of this study was to evaluate the within- and between-session reliability of using a laser device (LAVEG) to objectively quantify deceleration ability of rugby players from a maximal sprint task. It was hypothesised that stopping distance (distance to 0% of velocity at 15 m of the prior maximal 15 m sprint) and distances to specified percentages (75%, 50% and 25%) of velocity at 15 m of the maximal sprint would have acceptable reliability both within and between sessions.

## 2. Materials and Methods

### 2.1. Subjects

Twenty male amateur rugby union players (age: 31.8 ± 10.5 years, height: 1.84 ± 0.07 m, body mass: 99.8 ± 20.4 kg) who were free from injury were recruited to take part in the present study. All subjects trained and played rugby union regularly (minimum once per week) for at least one year preceding the experiment. The aims and details of the study and what participation in the study would involve were explained to each subject before completion of the written informed consent forms. Institutional (University of Salford) ethical approval (ethics number: HST1617-24) was granted before subject recruitment began.

### 2.2. Design

The reliability and sensitivity of the LAVEG (LAser VElocity Guard— LDM 300C, Jenoptik, Jena, Germany) deceleration test was assessed using a repeated-measures design. Three measurements were taken on two separate occasions, seven days apart. Subjects were asked to perform three maximal, 15 m sprints from a standing start, followed by an immediate deceleration to a complete stop. This distance was chosen because it is a common, short sprint distance in rugby union [15]. The data were then extrapolated and velocity at the 15 m mark was recorded and subsequent distance at 75%, 50%, 25% and 0% of the recorded velocity at 15 m was then recorded for both testing sessions. Within- (within three trials of each session) and between- (mean of each session) sessions reliability were examined.

### 2.3. Procedures

**Figure 1:**
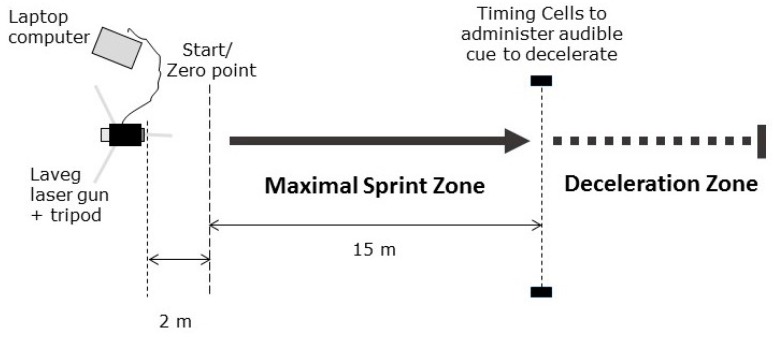
The deceleration test layout.

Subjects attended the testing field on three separate occasions. The first session ensured that each subject completed a familiarisation session in which the procedure was conducted before data were recorded. This was necessary due to prior pilot work, whereby a definitive learning effect was observed from session one to session two. Height was recorded to the nearest 0.01 m using a stadiometer (Harpenden, Burgess Hill, UK) and body mass was measured to the nearest 0.1 kg using digital scales (Salter, Bilston, UK). Prior to each testing session, the LAVEG was placed 2 m behind the start of the 15 m sprint track and a zero-point was calibrated at the start of the 15 m sprint (Figure 1). At each testing session, subjects performed a standardised warm up before testing was undertaken. This warm-up involved a typical pre-game warm-up routine incorporating a low-intensity jogging (10 min) sprint and low intensity plyometric drills (i.e., high-knee marching, running, skipping over 20 m), short sprint (20 m) and change of direction drills, increasing intensity of each effort (e.g., 50%, 75% and 100%).

Each testing session included a familiarisation task of three submaximal sprint and stops of increasing intensity (50% maximum intensity/ 70% maximum intensity/ 90% maximum intensity). Subjects were then asked to perform three maximal 15 m sprints. Upon hearing the audible cue at the 15 m mark they were instructed to decelerate and stop as quickly as possible and remain in the stop position for a minimum of 3 s. Two minutes’ rest was instructed between each trial to allow a full recovery. Measures of instantaneous velocity were recorded by the LAVEG. The same investigator (J.A.) performed all measurements with the LAVEG throughout both testing sessions. During the manual operation of the LAVEG within each trial the investigator focused the crosshair of the viewing lens on the lower torso (small of the back) throughout each trial.

The testing process was then repeated seven days later, at the same time of day (±1 h) and under similar environmental conditions to control for circadian variation and environmental influences on performance [16]. Each subject was instructed to wear the same shoes to control for shoe-surface interface between sessions. Subjects were instructed to refrain from strenuous physical activity in the 48 hours prior to testing and to ensure they were appropriately hydrated and had consumed a normal dietary intake before each testing session. These controls were asserted to minimise the influence of extraneous variables on their sprint performance, thus enhancing the internal validity and reliability of this study.

The LAVEG measured the positional information of each participant at 100 Hz. This information was then used to produce an instantaneous velocity profile for each subject. The data were smoothed using a 51-point moving average at intervals of 0.5 s to remove excess noise, and then extrapolated from the LAVEG software to Microsoft Excel. Velocity at 15 m (m·s^−1^) was recorded and quartiles (75%, 50% and 25%) of this velocity were calculated for each trial. Distance relative to the 15 m mark to the point where each subject in each trial reached 75%, 50%, 25% and 0% (stopping distance) of velocity at 15 m was recorded from the “distance/velocity” trace on the LAVEG software (Version 3.9, Jenoptik, Jena, Germany).

### 2.4. Statistical Analysis 

Data were collected over two sessions and statistically analysed using SPSS software (version 23.0, SPSS, Inc., IL, USA). Descriptive statistics (mean ± standard deviation, 95% confidence intervals) were reported for both sessions. The data were explored for normality using a Shapiro–Wilks test. Bland–Altman plots were used to inspect for the presence of heteroscedasticity of data between sessions. Intraclass correlation coefficient’s (two-way mixed effect model with absolute agreement) were determined for within- and between-session data. Standard error of measurement (SEM = SD_POOLED_ × √1 − ICC), smallest detectable difference (SDD = 1.96 × √2 × SEM) and coefficient of variation (SD/mean × 100), were determined for within session (between three trials) and between sessions. ICCs were considered excellent if ICC > 0.8 [17] with minimum acceptable reliability determined with an ICC > 0.7 and CV < 15% [18]. SEM and SDD were represented relative to mean values as well as absolute.

## 3. Results

### 3.1. Within-Session Reliability

The descriptive data for both sessions are reported in Table 1. Normality was confirmed for all variables using a Shapiro–Wilks test. No significant differences (*p* > 0.05) were observed between trials during session one and two for velocity at 15 m, and distances to 75%, 50%, 25% and 0% (stopping distance) of 15 m velocity. Within-session reliability was shown to be moderate to good across each testing session, with complete stopping distance (distance at 0% of 15 m velocity) shown to have excellent reliability (>0.8 ICC) in both testing sessions. Within-session CVs met minimal acceptable variation of <15% with the exception of distance to 75% and 50% of 15 m velocity in session two (Table 1).

### 3.2. Between Session Reliability

**Figure 2:**
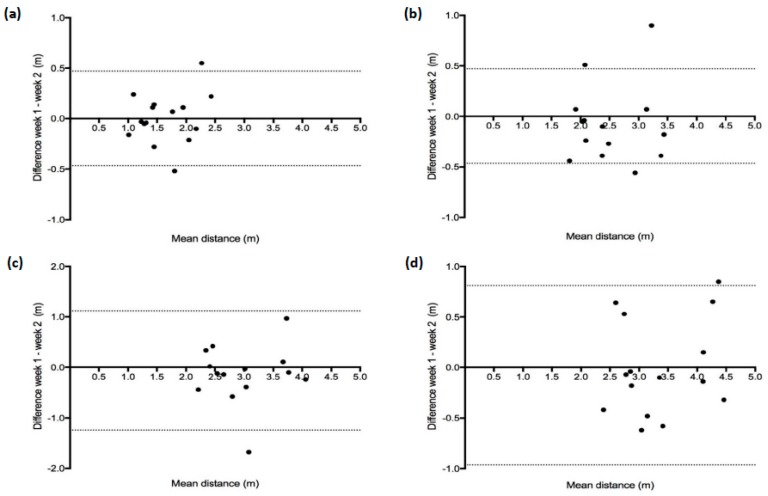
Bland–Altman analysis of the differences between week 1 distance and week 2 distance covered at quartiles of 15 m sprint velocity; 75% (**a**), 50% (**b**), 25% (**c**) and 0% (**d**) velocity vs. the mean distance for each quartile.

Table 2 shows between-session reliability for the deceleration test. No significant differences (*p* > 0.05) were observed between sessions for velocity at 15 m, and distances to 75%, 50%, 25% and 0% (stopping distance) of 15 m velocity. ICCs demonstrated excellent reliability (ICC ≥ 0.79) between sessions for all variables, whilst between-session CVs met minimal acceptable variation of <15% with the exception of distance to 75% of velocity at 15 m (Table 2). Bland–Altman plots in Figure 2 reveal the presence of homoscedasticity between weekly observations.

## 4. Discussion

The aim of this study was to evaluate the within- and between-session reliability of using a laser device (LAVEG) to determine deceleration ability from a maximal 15 m sprint. Stopping distance (0% of velocity at 15 m) demonstrated excellent within- and between-session relative reliability. Furthermore, between-session reliability was excellent for distances to achieve specific percentage of velocity decrement (ICC ≥ 0.79), confirming the study hypotheses. Within-session reliability was moderate, but good for overall stopping distance (ICC = 0.64–0.83). Reasonable between-session absolute reliability was reported; suggesting a moderate sensitivity for stopping distance (distance to achieve 0% of 15 m velocity) with relative SDD of 9.11% for between sessions. Thus, further work is needed to perhaps improve the absolute reliability further to establish a greater level of sensitivity to monitor changes in athletes as a result of interventions.

Despite a lot of attention to methods to evaluate both change of direction speed and agility, little attention has been paid to develop methods in order to evaluate deceleration ability. Previous suggestions have ranged from counting the number of steps to stop from sprints of various intensity [11], and stopping distances following a sprint measured via video analysis [13] or tape measure [12]. No studies have examined the reliability of such deceleration tests or used the LAVEG to quantify stopping distance. The LAVEG has previously been proved as a reliable tool in measuring 10–50 m sprint times (ICC ≥ 0.922; CV = 1.9%–3.1%; SEM = 0.02 s) [19,20,21,22]. Furthermore, Poulos [20] reported maximum velocity during 50 m sprints to have low variation (CV = 1.9%), but greater variability was observed for distance of maximum velocity (CV = 18.2%). In addition, Bezodis et al. [23], reported that recorded velocities at distances >5 m have good agreement with a criterion method of determining centre of mass velocity from digitising high-speed video (bias ± error limits; 10 m: +0.16 ± 0.11 m·s^−1^; 30 m: +0.06 ± 0.13 m·s^−1^; 50 m: +0.08 ± 0.15 m·s^−1^). To the authors knowledge, this is the first study to use and examine the reliability of the LAVEG to assess deceleration ability. The results reveal that stopping distance from a 15 m maximal sprint has good to excellent within- and between-session reliability (ICC within = 0.82–0.83; ICC between = 0.91) and acceptable between-session absolute reliability (CV = 10.55%; SEM = 0.23 m (6.54%); SDD = 0.64 (9.11%)). Furthermore, reporting distances to specific percentages of velocity at the end of the 15 m sprint provided good relative reliability (ICC = 0.79–0.93) and acceptable absolute reliability (CV = 13.55%–15.63%; SEM = 0.12–0.32 m) between sessions, but much lower absolute within-session reliability (ICC = 0.62–0.73; CV = 12.33%–16.71%; SEM = 0.31–0.54 m); suggesting that overall stopping distance is the most reliable metric to quantify deceleration ability.

Although for most variables reported in the study the variation between trials met an acceptable level (<15%) with the exception of distance to achieve 75% and 50% of velocity at 15 m in session two. In general, a large variation in all four measures was evident within each session (CV = 10.51%–16.71%) and could be largely explained by the movement variation associated with the task and the intra-operator reliability of using the LAVEG. The ability to decelerate is a complex skill that involves integration of several muscular systems, control of multiple large joints and extremely high forces across multiple planes of movement when performed maximally [24], as in this instance. Team sport players are required to accelerate and decelerate multiple times throughout match play and training [2,3,4,5,25]. These accelerations and decelerations are triggered by any number of different situations; from evading opposition, to moving tactically to position themselves [1]. Each player presumably, has multiple pre-set strategies to perform this task to account for variation in approach speed, approach stride-length, team/opposition positioning, shoe-surface interface, dominant/ non-dominant foot-plant and timing in relation to each given stimulus during match play and training. Due to the ability level of the athlete population in this study, the assumption that movement variability is high can explain the typical variation (CV) in the reported data [26]. Further investigations with athletes competing at a higher level of competition should be completed to determine whether lower variation is indeed observed during this testing protocol.

The LAVEG requires manual operation by the tester to track a single point on the athlete’s torso (small of the back) during trials. Thus, intra-operator consistency could also account for some of the variation between trials. Further work should be carried out to explore typical inter-operator agreement (using two LAVEGs during one trial) to isolate the impact of operator influence on reliability when using this protocol.

Only one previous study [14] has used the LAVEG to quantify deceleration ability in athletes. This protocol involved measuring instantaneous velocity during acceleration and (immediate) deceleration efforts within set distances of 5, 10, 15 and 20 m determining maximum velocity, stopping distances and percentage of maximum velocity based on a prior 30 m sprint test. This approach was used with a view to set realistic conditions for acceleration–deceleration drills. The present study used a protocol based on previous deceleration studies [11,12,13]. However, a limitation of such a protocol is that it may not replicate the typical acceleration–deceleration efforts performed in sport, as evident with the protocol used by Graham-Smith et al. [14]. Furthermore, another disadvantage of the methods used in the current study is that it is assumed that athletes are achieving a close to maximum velocity (within the 15 m sprint) at 15 m, when in fact maximum velocity may well have been achieved prior to the 15 m mark (peak velocity session 1: 6.86 ± 0.48 m·s^−1^; session 2: 6.81 ± 0.5 m·s^−1^ vs. velocity at 15 m session 1: 5.36 ± 0.72 m·s^−1^; session 2: 5.41 ± 0.66 m·s^−1^). Although an audible bleep was used in this study by virtue of the athletes running through a timing gate as a command to begin deceleration, athletes may still ‘anticipate’ the deceleration prior to the audible bleep as they approach the timing cells. Utilising acceleration–deceleration efforts [14] may avoid this as acceleration and deceleration (stopping) distances are determined in relation to the maximum velocity achieved during the effort. Future work should quantify the reliability of the acceleration–deceleration protocol [14] and explore the relationships between these different assessment methods. Future work should also compare the agreement between using the LAVEG to determine stopping distances and using video and tape measure methods previously reported [12,13]. This would evaluate whether alternative methods to determine stopping distance are valid for coaches who do not have access to LAVEG technology.

## 5. Conclusions

The findings of the present study suggest that using laser devices such as the LAVEG is a reliable method to quantify stopping distance from a 15 m sprint task and offers a solution to quantify deceleration ability in team sport athletes. The results further show that ‘stopping distance’ has a greater level of relative and absolute reliability than determining deceleration distances to certain percentage velocity decrements and, thus, should be the sole metric to determine deceleration ability. Based on the reported data, changes in stopping distance of >0.64 m (9.11%) identify meaningful changes in response to training or competition for amateur rugby players. Future research is needed to explore the use of alternative versions of the test (i.e., altered sprint distances or using acceleration–deceleration efforts) and compare the agreement of measuring ‘stopping distance’ from the LAVEG against video methods previously used.

## Figures and Tables

**Table 1:** Within-session reliability of sprint deceleration test for sessions 1 and 2.

Session 1						
Variable	Mean ± SD	95% CI	ICC	CV	SEM (Abs)	SEM (Rel)
			(95% CI)	(95% CI)		
Velocity at 15 m (m·s^−1^)	5.36 ± 0.72	5.67–5.04	0.82	5.11%	0.32	6.02%
			(0.66–0.92)	(3.53–6.69)		
Distance at 75% of 15 m velocity (m)	1.69 ± 0.48	1.90–1.48	0.73	14.55%	0.31	18.34%
			(0.53–0.87)	(10.04–19.06)		
Distance at 50% of 15 m velocity (m)	2.78 ± 0.68	3.08–2.48	0.68	11.98%	0.47	16.76%
			(0.45–0.84)	(8.27–15.69)		
Distance at 25% of 15 m velocity (m)	3.07 ± 0.76	3.40–2.74	0.80	12.33%	0.39	12.60%
			(0.63–0.91)	(8.51–16.15)		
Distance at 0% of 15 m velocity (m)	3.58 ± 0.80	3.93–3.23	0.83	10.60%	0.37	10.43%
			(0.64–0.93)	(7.32–13.88)		
**Session 2**						
Velocity at 15 m (m·s^−1^)	5.41 ± 0.44	5.70–5.12	0.83	5.48%	0.30	5.48%
			(0.68–0.92)	(3.78–7.18)		
Distance at 75% of 15 m velocity (m)	1.76 ± 0.44	1.95–1.57	0.83	16.71%	0.23	13.06%
			(0.69–0.92)	(11.53–21.89)		
Distance at 50% of 15 m velocity (m)	2.60 ± 0.60	2.86–2.34	0.68	15.93%	0.45	17.25%
			(0.47–0.84)	(10.99–20.87)		
Distance at 25% of 15 m velocity (m)	3.14 ± 0.66	3.43–2.85	0.64	14.77%	0.54	17.08%
			(0.41–0.82)	(10.19–19.34)		
Distance at 0% of 15 m velocity (m)	3.46 ± 0.73	3.78–3.14	0.82	10.51%	0.38	11.08%
			(0.66–0.92)	(7.25–13.77)		

Note: SD = standard deviation; CI = confidence interval; ICC = intraclass correlation coefficient; CV = coefficient of variation; SEM = standard error of measurement; SDD = smallest detectable difference; Abs = absolute; Rel = relative.

**Table 2:** Between-session reliability of sprint deceleration test.

Variable	ICC	CV	SEM (Abs)	SEM (Rel)	SDD (Abs)	SDD (Rel)
	(95% CI)	(95% CI)				
Velocity at 15 m (m·s^−1^)	0.97	1.87%	0.12	2.21%	0.33	6.14%
	(0.93–0.99)	(2.45–1.29)				
Distance at 75% of 15 m velocity (m)	0.93	15.63%	0.12	7.04%	0.34	13.19%
	(0.81–0.98)	(10.79–20.47)				
Distance at 50% of 15 m velocity (m)	0.88	13.96%	0.22	8.23%	0.61	14.13%
	(0.67–0.96)	(9.63–18.29)				
Distance at 25% of 15 m velocity (m)	0.79	13.55%	0.32	10.45%	0.90	11.97%
	(0.41–0.92)	(9.35–17.75)				
Distance at 0% of 15 m velocity (m)	0.91	10.55%	0.23	6.54%	0.64	9.11%
	(0.75–0.97)	(7.28–13.82)				

CI = confidence interval; ICC = intraclass correlation coefficient; CV = coefficient of variation; SEM = standard error of measurement; SDD = smallest detectable difference; Abs = absolute; Rel = relative.
